# Mechanical energetics and dynamics of uphill double-poling on roller-skis at different incline-speed combinations

**DOI:** 10.1371/journal.pone.0212500

**Published:** 2019-02-22

**Authors:** Jørgen Danielsen, Øyvind Sandbakk, David McGhie, Gertjan Ettema

**Affiliations:** Centre for Elite Sports Research, Department of Neuromedicine and Movement Science, Faculty of Medicine and Health Sciences, Norwegian University of Science and Technology, Trondheim, Norway; Universita degli Studi di Verona, ITALY

## Abstract

**Objectives:**

The purpose of this study was to investigate the effect of different incline-speed combinations, at equal external power outputs, on the mechanics and energetics of the double-poling (DP) technique in cross-country skiing.

**Methods:**

Fourteen elite male cross-country skiers performed treadmill DP on roller-skis at low, moderate, and high mean external power outputs (P_mean_) up a shallow incline (5%, INC5), at which DP is preferred, and up a steep incline (12%, INC12), at which DP is not preferred. Speed was set to produce equal P_mean_ at both inclines. From recorded kinematics and dynamics, arm power (P_arm_) and trunk+leg power (P_T+L_) were derived, as were pole propulsion power (P_pole_) and body mechanical energy perpendicular to the treadmill surface (E_body⊥_).

**Results:**

Over a locomotion cycle, the arms contributed 63% to P_mean_ at INC5 but surprisingly only 54% at INC12 (*P*<0.001), with no effect of P_mean_ (*P* = 0.312). Thus, the trunk and legs contributed substantially to P_mean_ both at INC5 (37%) and INC12 (46%). At both inclines, P_T+L_ generation during the swing phase increased approximately linearly with P_mean_, which increased E_body⊥_. Within the poling phase, ~30–35% of the body energy which was developed during the preceding swing phase was transferred into propulsive pole power on both inclines. At INC5, the amount of negative P_T+L_ during the poling phase was larger than at INC12, and this difference increased with P_mean_.

**Conclusions:**

The considerable larger amount of negative P_T+L_ during poling at INC5 than at INC12 indicate that the legs and trunk generate more power than ‘necessary’ during the swing phase and thus must absorb more energy during the poling phase. This larger surplus of P_T+L_ at INC5 seems necessary for positioning the body and poles so that high P_arm_ generation can occur in a short time. At INC12, less P_arm_ is generated, probably due to less advantageous working conditions for the arms, related to body and pole positioning. These incline differences seem linked to shorter swing and longer poling times during steep uphill DP, which are due to the increased influence of gravity and slower speed at steep inclines.

## Introduction

Double poling (DP) is one of the main techniques in classical style cross-country (XC) skiing, and its usage and importance during training and races has increased during the last two decades [1–3]. DP is mainly used on flatter parts of a course, but some skiers may use DP exclusively during entire races, even those containing steep uphill sections (>10–12%) [4]. This occurs even though studies have shown that on inclines steeper than 8–9%, skiers prefer to use the diagonal stride technique rather than DP [5, 6]. Choosing DP exclusively eliminates the necessity for grip waxing and thus reduces the power lost to gliding friction on the flatter and downhill sections of a course.

In DP, propulsive forces are generated solely through the poles during symmetrical and synchronous pole movements during the poling phase, while the skis glide continuously. During the swing phase, after the poles are lifted from the surface and are being repositioned, the skier’s forward velocity slows due to friction. Although all propulsive forces are directed through the poles, DP is a whole-body movement in which involvement of the legs and trunk is important for optimal generation of pole forces during the subsequent poling phase [3, 7–10].

During the swing phase, the legs generate power which results in a raising of the body centre of mass (CoM), thus increasing the body’s gravitational potential energy level. Immediately prior to and during a portion of poling, the body is rapidly lowered as if the skier is ‘falling onto the poles’. This strategy, employing more of the large muscle mass in the legs and core is increasingly used at faster velocities [11, 12]. Thus, the relative power contribution by the legs increases with enhanced DP intensity [10]. During the poling phase, a considerable part of the instantaneous pole propulsion power (P_pole_) may originate from a transfer of body mechanical energy (E_body_), i.e., from work done at the legs in the preceding swing phase. If this mechanism is removed, for example by minimizing the involvement of the legs, power output and performance decreases [8, 13].

**Fig 1:**
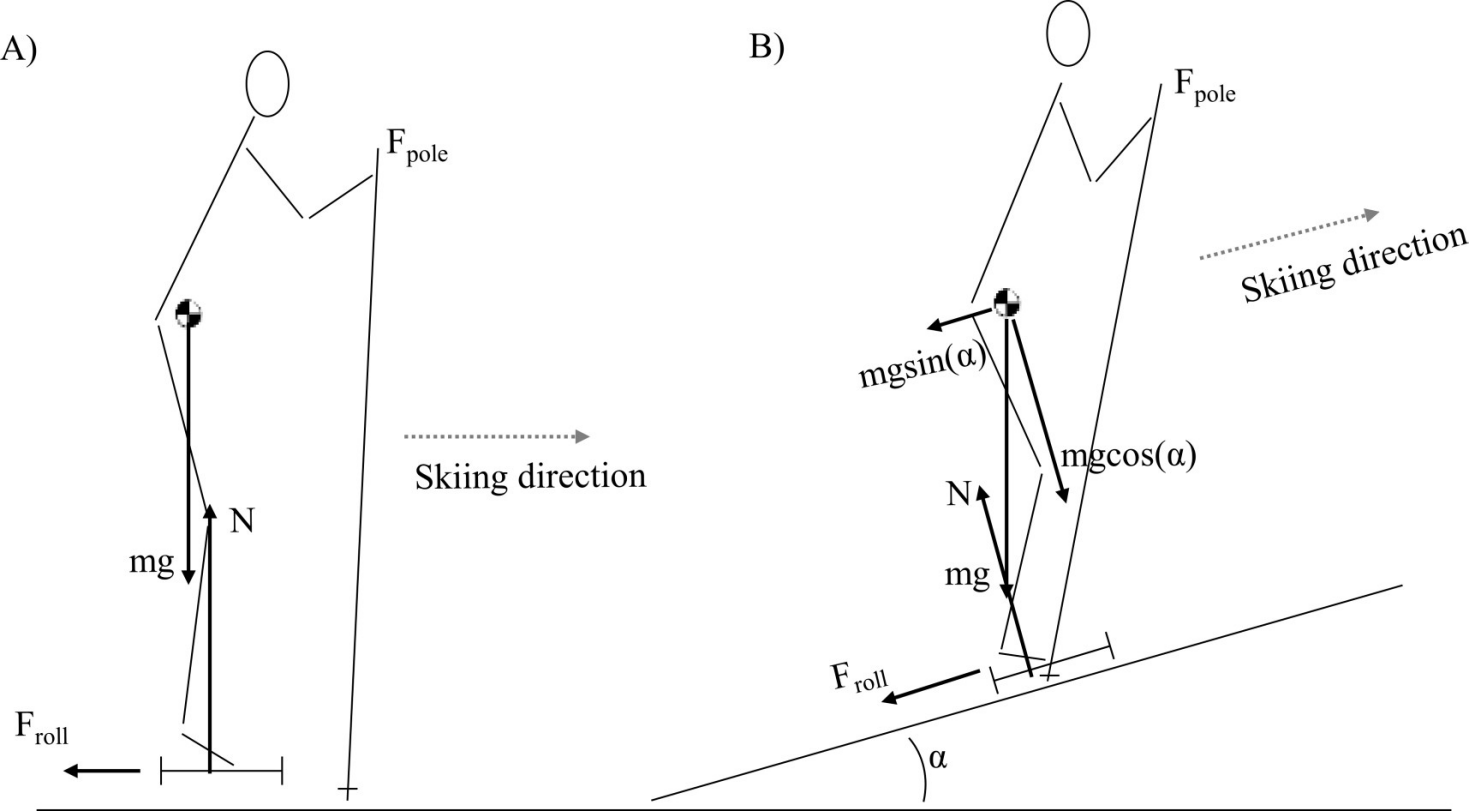
**Typical body and pole positioning at pole plant during DP on the level (A) and on steep incline (B)**. The gravity component parallel to the surface, mgsin(α), increases with incline and induces greater speed losses whenever the poles are off the ground. If the same body positioning relative to the level ground were to be realized on an incline, external forces would likely lead to an unstable situation, e.g., the force of gravity (mg) would generate a moment at the base of support which would be hard to counteract by N or F_pole_. Therefore, skiers alter body positioning on a slope compared to level skiing.

Most studies dealing with these mechanical aspects of DP investigated this technique during level roller or on-snow skiing or in ergometer DP [7–9, 11, 13, 14]. However, on steeper inclines, the component of gravity parallel to the surface becomes larger. In addition, gravity generates a larger moment about the base of support (Fig 1). These different boundary conditions on a slope have implications for movement execution. As a result, skiers reduce swing times and increase poling times [1] to reduce the greater forward (parallel) speed loss induced during the swing phase at steeper inclines. Reducing swing time leads to less time available to reposition the body and poles optimally before the subsequent poling phase, challenging effective use of the DP technique [1, 14]. Moreover, at steeper inclines, skiers change body and pole positioning. The lower-extremity joints become more flexed, the highest CoM position occurs closer in time to pole plant and the poles are planted closer to the feet [1]. Thus, there is likely less to gain from increasing perpendicular potential energy during the swing phase on steep uphill slopes. This should increase the work demand by the arms at steep inclines, and may play a role in explaining why skiers prefer DP on shallow but not steep inclines [6]. One may therefore hypothesize that the capacity to use the legs as a major source of power generation throughout the DP cycle is reduced at steeper inclines. However, Stöggl and Holmberg [1] found increased flexion-extension range of motion (ROM) in the lower-extremities throughout the cycle, and less ROM and a more upright trunk in uphill compared to level DP. This may indicate that leg power is even greater on an incline. In that study, however, external power (P_mean_) was also substantially greater on the uphill, obscuring the effect of incline separate from P_mean_. No studies have yet estimated the power output of the arms and legs in uphill DP at different inclines but at equal external power.

Overall, the relative contribution or involvement of the legs increases with increasing external power both during ergometer DP [10] and on-snow skiing DP on level terrain [12]. However, it remains to be examined whether this relationship holds true for DP on steeper inclines. Moreover, further elucidation is needed regarding differences in the dynamics of the execution of the DP technique between inclines. Therefore, the purpose of this study was to examine the dynamics of DP at different incline-speed combinations. Given different boundary conditions (Fig 1), it was hypothesised that the relative contribution from the legs is reduced from a shallow to a steeper incline. Such a finding may support the notion that effective execution of the DP technique is hampered at steep inclines where DP is no longer the preferred technique.

## Methods

### Participants

Fourteen male Norwegian national and elite level XC skiers (mean ± SD: age 23.7 ± 2.6 yrs, height 1.83 ± 0.05 m, body mass 76.1 ± 6.6 kg) volunteered to take part in this study. They were all familiar with treadmill roller-skiing from daily training and testing routines. Before providing written informed consent, all participants were verbally informed about the nature of the study. The right to withdraw at any point was explicitly stated. The study was registered at and approved by the Norwegian Social Science Data Services, and the study was conducted in accordance with the Declaration of Helsinki.

### Experimental design

The skiers warmed up for ~15 minutes at low and moderate intensities, performing DP roller-skiing on a treadmill at a variety of speeds and inclines (including those used in the main experiments), in addition to a few short high-intensity bouts. All skiers used the same pair of roller skis, and the warm-up period ensured that the wheels and bearings reached proper temperatures.

The main experiments consisted of DP roller-skiing for ~100 s at low, moderate, and high P_mean_ on two different inclines: 5% (INC5) and 12% (INC12). Between each condition the skiers rested for ~2 min, and the order of each incline-speed combination was randomized. In a recent study based on the same data as the present study, the same group of skiers preferred to use DP at INC5 but not at INC12, corresponding to an enhanced sense of effort in the arms and reduced efficiency at INC12 [6]. Speeds were set to elicit similar P_mean_ at the two inclines, estimated based on average body mass. Thus, all skiers individually generated a similar P_mean_ on both inclines. The P_mean_ generated by each skier corresponded to approximately 55%, 70%, and 80% of maximal rate of oxygen consumption at low, moderate, and high, respectively, while the mean difference between each P_mean_ was ~47 W (range: 41–57 W). The skiers were told to remain in approximately the same position on the treadmill and to maintain self-selected cycle rates stable. Kinetics and kinematics were then recorded during the last ~75 s.

### Instruments and materials

Roller-skiing was performed on a 5 x 3 m motor driven treadmill (Forcelink Technology, Culemborg, The Netherlands). All skiers used the same pair of classical roller skis with resistance category 2 (IDT Sports, Lena, Norway). The skiers used poles of preferred length (~83% of body height), available in 5 cm increments (Madshus UHM 100, Biri, Norway). Special carbide tips were used ensuring good grip on the surface of the treadmill belt covered with non-slip rubber. All skiers were secured with a safety harness connected to an emergency brake. Before and after the experiments, the coefficient of rolling resistance (μ) of the roller skis was determined three times by a towing test previously described [15]. The mean value of μ both before and after the experiments was 0.018 ± 0.001.

### Kinetics

The resultant pole force of each pole was measured with CDF Miniature Button Load Cells (diameter, 15 mm; height, 8 mm; capacity, 2 kN; non-linearity, < .5%; weight, 10 g; Applied Measurements LTD, Aldermaston, Berkshire, UK) [6]. These were placed on top of an aluminium tube (50 g), directly mounted at the top of and inside the pole tube. A small 8 mm diameter ball was located between the load cell and the aluminium tube, minimizing possible cross-talk between forces directed along the pole and forces related to squeezing, bending or rotation of the hand grip. The pole forces were calibrated against a force platform (Kistler 9286BA, Kistler Instruments, Winterhur, Switzerland), on which several poling-like actions were performed, and the maximal error during peak force was ~10 N. Pole force data was sampled at 1500 Hz and recorded via a telemetric system (TeleMyo DTS, Noraxon, Scottsdale, AZ, USA) connected to Qualisys Track Manager software (Qualisys AB, Gothenburg, Sweden).

### Kinematics

Nine infrared Oqus 400 cameras (Qualisys AB) were placed around the treadmill to capture three-dimensional position characteristics of passive spherical reflective markers (ø 14 mm) at a sampling frequency of 250 Hz. The volume of measurement was calibrated according to the manufacturer’s specifications. The same researcher placed all markers on all skiers’ anatomical landmarks bilaterally using double-sided tape (3M, St. Paul, MN, USA). These landmarks were on the ski boot on the head of the fifth metatarsal, the ski boot on top of the lateral malleolus (ankle), the lateral femoral epicondyle (knee), the greater trochanter (hip), the lateral end of the acromion process (shoulder), the lateral humeral epicondyle (elbow), and the styloid process of ulna (wrist). These markers defined 11 body segments: foot, shank, thigh, upper arm, forearm, and trunk. Two markers were placed on each pole, one marker ~5 cm below the grip handle and one marker on the lateral side of the carbide pole tip. Two markers were placed on each ski, one marker 1 cm behind the front wheel and one marker 1 cm in front of the back wheel. Two markers were placed on the treadmill in alignment with belt movement direction, continuously tracking treadmill inclination. The Qualisys Track Manager software synchronized and stored both kinetics and kinematics, and further analysis was performed in Matlab (R2016b, Mathworks Inc., Natick, MA, USA).

### Data analysis

Force and marker position data were low-pass filtered (8^th^ order, zero-lag, Butterworth filter) with the same cut-off frequency of 15 Hz [16, 17]. Inertial properties of body segments were estimated using equations based on segment lengths and body mass [18]. The mass of the skis and poles were added to the mass of the feet and forearms, respectively. Segment lengths were determined from the average of marker coordinates over the entire period of analysis. CoM of the forearms and trunk was adjusted to include the hands and head, respectively. Linear and angular velocities and accelerations of the limb segments and the whole-body CoM were obtained by numerical differentiation of position data with respect to time. The poling phase was defined as the period when the poles were in contact with the treadmill belt. Belt contact was defined as the period when movement direction and velocity magnitude of the markers on the pole tips and the treadmill belt were (close to) identical. Consequently, swing time was defined as the period when the poles were off the belt.

In indoor (no wind) treadmill DP at steady-state speeds on an inclination, the skier has to generate P_mean_ to overcome power losses to rolling resistance and gravity:

$$P_{mean}=v(m\;g\;\sin\;\alpha +(m\;g\;\cos\;\;\alpha -{\bar{F}}_{{pole}_{⊥}})µ)$$ (1)

where v is the velocity of the treadmill belt, m is body mass including equipment, g is gravitational acceleration (9.81 m·s^-2^), ${\bar{F}}_{{pole}_{⊥}}$ is the cycle mean perpendicular component of pole force and α is angle of treadmill inclination. In DP, both the perpendicular and parallel velocities of the CoM fluctuates considerably within one movement cycle, and the total instantaneous muscle power generated to overcome rolling resistance, gravity, and to induce changes in body mechanical energy varies at different inclines and speeds. An instantaneous power equation relating power production to dissipation [19] in treadmill roller-skiing can be written as:

$$P_{tot}=d\sum E_{seg}/dt+P_{roll}$$ (2)

where P_tot_ is the instantaneous total muscle power output, ∑E_seg_ is the sum of the translational and rotational kinetic and potential energy of the individual body segments (E_seg_), and P_roll_ is the power against rolling resistance. E_seg_ was calculated as:

$$E_{seg}=\frac{1}{2}{m_{seg}v_{seg}}^{2}+m_{seg}gh_{seg}+\frac{1}{2}I_{seg}{\omega_{seg}}^{2}$$ (3)

where m is segment mass (kg), v is the instantaneous absolute segment velocity, and h the instantaneous segment height, in the coordinate system moving with treadmill belt speed, I is segment moment of inertia (kg·m^2^), and ω is segment angular velocity (rad·s^-1^) [20]. P_roll_ was estimated as

$$P_{roll}=(mg\;\cos\;\alpha -{\bar{F}}_{{pole}_{⊥}})\mu v_{{CoM}_{∥}}$$ (4)

where $v_{{CoM}_{∥}}$ is the instantaneous velocity of CoM parallel to the belt.

P_tot_ includes the power against rolling friction and gravity and the rate of energy changes associated with body movements in goal-direction (P_∥_) as well as the rate of change of energy associated with movements perpendicular to goal-direction (${\dot{E}}_{{body}_{⊥}}$, i.e., the rate of ‘internal’ energy changes, mainly potential and kinetic energy changes related to (perpendicular) body lowering and raising). Averaged over a cycle at steady-state speeds, the mean change of ${\dot{E}}_{{body}_{⊥}}$ is zero, while the mean change in P_∥_ equals the power associated with overcoming gravity plus rolling resistance, since net acceleration is zero at constant speeds. The fluctuation of ${\dot{E}}_{{body}_{⊥}}$ in relation to P_pole_ is presumably dependent on different incline-speed combinations and indicates the transfer of $E_{{body}_{⊥}}$ to P_pole_ [9]. ${\dot{E}}_{{body}_{⊥}}$ can be approximated as:

$${\dot{E}}_{{body}_{⊥}}=P_{tot}-P_{∥}$$ (5)

while P_∥_ can be estimated using Eq 1:

$$P_{∥}=v_{{CoM}_{∥}}(mg\;\sin\;\alpha +(mg\;\cos\;a-{\bar{F}}_{{pole}_{⊥}})\mu +ma_{{CoM}_{∥}})$$ (6)

where $a_{{CoM}_{∥}}$ is the acceleration of CoM in goal-direction (i.e., parallel to the treadmill belt) [6].

P_pole_ was calculated as:

$$P_{pole}=F_{pole}\;V_{CoM}\;\cos\;\beta$$ (7)

where F_pole_ is the pole force vector (the direction of which was determined from the pole markers), V_CoM_ is the CoM velocity vector (relative to treadmill belt speed), and *β* is the angle between these two vectors (e.g., [6, 21]). It should be noted that as the poles hit the ground, the point of force application does not move (in the coordinate system moving with treadmill belt speed). Thus, P_pole_ is not a true measure of power as defined in mechanics, i.e., the dot product of force and velocity. However, the pole force vector is considered to propel the CoM, as in e.g., walking and running [21]. Averaged over a cycle, P_pole_ should be equal to P_mean_ and cycle average P_tot_. However, instantaneous P_pole_ and P_tot_ will not necessarily be equal, because P_tot_ contains both ‘external’ and ‘internal’ power. For example, repositioning and raising of the body during the swing phase demands positive P_tot_ while P_pole_ is zero.

Inverse dynamics [22] were used to calculate the net (sagittal plane) joint moments developed at the shoulder and elbow joints, and both shoulder and elbow joint power were calculated as the dot product of joint moment and joint angular velocity. The sum of elbow and shoulder power was defined as arm power (P_arm_). Because of considerable flexion-extension movements within the trunk and a lack of measurements of ski forces and point of application, inverse dynamics were deemed accurate and performed only for the upper extremity. In linked segment modelling, the summed joint power must equal P_tot_ (Eq 2) [19], but since only P_arm_ was calculated, the residual of P_tot_ and P_arm_ was considered to be power originating at the trunk and legs (P_T+L_), in a similar way as in [10, 23].

In order to investigate how the relative work load and power contribution from P_arm_ and P_T+L_ depends on P_mean_ and incline, a similar method was adopted from previous literature [10, 24, 25]. The time traces of P_tot_, P_arm_, and P_T+L_ were integrated over the duration of the cycle, the poling phase, and the swing phase, giving the work done in the respective phases. These work values were then divided by cycle time, poling time, and swing time to give average power values ($\bar{P}$) for the respective time periods. Furthermore, P_arm_ and P_T+L_ were time integrated over their respective positive and negative periods, independently for the poling and swing phases. The sum of all positive and negative work values for the poling and swing phase was then divided by poling time and swing time, respectively. This yielded average positive ($\bar{P}$^+^_arm_ and $\bar{P}$^+^_T+L_) and negative ($\bar{P}$^-^_arm_ and $\bar{P}$^-^_T+L_) arm and trunk+leg power in the poling and swing phases.

All data were time normalized for each participant for each cycle and averaged over ~20 cycles for each condition, and group mean ± 95% confidence interval (CI) curves were obtained by averaging across all participants.

### Statistical analysis

All data were checked for normality by visual inspection of normal Q-Q plots and histograms and are presented as means ± 95% CI. To test for interaction effects between P_mean_ and incline, two-way repeated measures ANOVA (2 inclines x 3 external powers) were performed. In the case of significant interaction effects, tests for simple main effects of P_mean_ at each incline were performed by one-way repeated measures ANOVA, where location of local differences were found using Fisher least significant difference while paired t-tests evaluated differences between incline at each P_mean_. In the case of non-significant interaction effects, two-way repeated measures ANOVA (without interaction term) was employed to test for main effects of incline and P_mean_, with Fisher least significant difference used post-hoc to locate differences between P_mean_ (for the pooled inclines). Statistical significance was set at p<0.05 and all statistical tests were performed using SPSS version 24 (IBM Inc., Armonk, NY, USA) and Microsoft Excel (Office 2016, Microsoft Corporation, Redmond, WA, USA).

## Results

### Cycle characteristics

**Fig 2:**
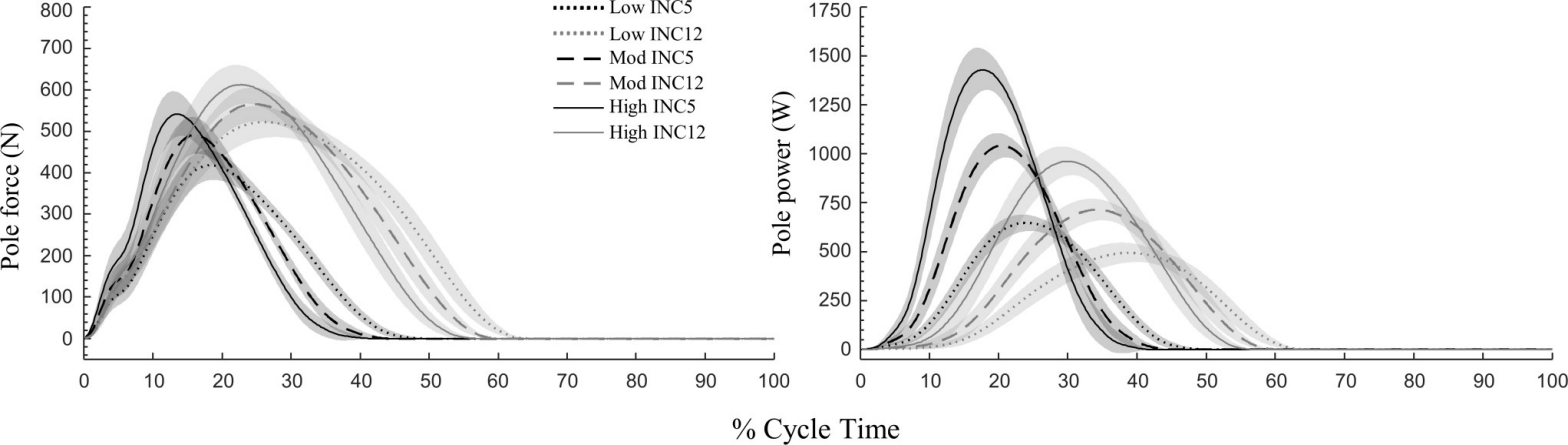
Pole force and pole power against normalized time during uphill DP at shallow (INC5, 5%) and steep (INC12, 12%) inclines. Lines are mean and shaded areas indicate 95% CI [N = 14].

P_mean_ was similar on both inclines and increased by ~47 W between each P_mean_ (p<0.001; Table 1). Cycle mean P_tot_ and mean pole power was approximately equal to P_mean_, indicating good measurement accuracy. The skiers used a faster cycle rate at INC12, and thus work per cycle was greater at INC5 compared to INC12. At both inclines, absolute and relative poling time decreased with increasing external power. Poling time was longer whereas swing time was shorter at INC12 than at INC5. The relative poling time ranged from 44% to 36% at low to high P_mean_ at INC5, and from 60% to 52% at INC12. While mean P_pole_ was close to P_mean_ at both inclines, peak P_pole_ was much larger at INC5 compared to INC12, the difference increasing with P_mean_ (interaction effect p<0.001; Table 1; Fig 2). This large difference is mostly due to the faster treadmill speed and thus instantaneous CoM velocities at INC5. Peak pole force and time to peak pole force was greater at INC12 (p<0.001; Table 1; Fig 2). The pole force was directed more in the backwards direction at pole plant at INC12 compared to INC5, but less backwards at pole off (both p<0.001).

**Table 1:** Variables associated with uphill double-poling on roller-skis at shallow (INC5, 5%) and steep (INC12, 12%) inclines at increasing external power outputs. Values are means ± 95% CI [*N* = 14].

Variables	Power output	Incline			Statistics		
					Interaction	Incline	Power output
					p ($\eta_{p}^{2}$)	p ($\eta_{p}^{2}$)	p ($\eta_{p}^{2}$)
		5%		12%			
Speed (m·s^-1^)	Low	2.58		1.33			
	Mod	3.43		1.77			
	High	4.31		2.21			
P_mean_ (W)	Low	142 ± 7	^BC^	143 ± 7	0.520 (0.05)	0.294 (0.08)	<0.001 (0.99)
	Mod	188 ± 9	^AC^	190 ± 9			
	High	237 ± 12	^AB^	238 ± 11			
Mean P_o_ (W)	Low	141 ± 7	^BC^	143 ± 7	0.440 (0.06)	0.255 (0.10)	<0.001 (0.99)
	Mod	188 ± 9	^AC^	190 ± 9			
	High	236 ± 12	^AB^	237 ± 11			
Cycle rate (Hz)	Low	0.84 ± 0.03	^BC^	0.88 ± 0.06	0.125 (0.15)	<0.01 (0.43)	<0.001 (0.63)
	Mod	0.87 ± 0.03	^AC^	0.93 ± 0.04			
	High	0.89 ± 0.02	^AB^	0.97 ± 0.03			
Poling time (s)	Low	0.53 ± 0.02	^BC^	0.70 ± 0.06	0.124 (0.15)	<0.001 (0.90)	<0.001 (0.86)
	Mod	0.46 ± 0.02	^AC^	0.61 ± 0.03			
	High	0.40 ± 0.02	^AB^	0.54 ± 0.03			
Swing time (s)	Low	0.67 ± 0.02	^BC^	0.46 ± 0.03	0.429 (0.06)	<0.001 (0.93)	<0.001 (0.51)
	Mod	0.70 ± 0.03	^AC^	0.47 ± 0.02			
	High	0.72 ± 0.03	^AB^	0.50 ± 0.02			
Poling time (% cycle time)	Low	44 ± 1	^BC^	60 ± 1	0.592 (0.04)	<0.001 (0.97)	<0.001 (0.94)
	Mod	40 ± 2	^AC^	56 ± 1			
	High	36 ± 1	^AB^	52 ± 2			
Mean pole power (W)	Low	143 ± 6^bc^		142 ± 7^bc^	<0.001 (0.58) F_(2,26)_ = 18		^5%^<0.001 (0.97) ^12%^<0.001 (0.99)
	Mod	195 ± 9^ac^^*^		187 ± 8^ac^			
	High	248 ± 11^ab^^*^		236 ± 10^ab^			
Peak pole power (W)	Low	660 ± 40^bc^^*^		527 ± 33^bc^	<0.001 (0.94) F_(2,26)_ = 191		^5%^<0.001 (0.98) ^12%^<0.001 (0.97)
	Mod	1059 ± 64^ac^^*^		738 ± 46^ac^			
	High	1469 ± 92^ab^^*^		1008 ± 63^ab^			
Peak pole force (N)	Low	425 ± 33^bc^^*^		534 ± 35^bc^	<0.001 (0.52) F_(2,26)_ = 14		^5%^<0.001 (0.90) ^12%^<0.001 (0.81)
	Mod	506 ± 39^ac^^*^		570 ± 37^ac^			
	High	562 ± 41^ab^^*^		626 ± 43^ab^			
Time to peak pole force (ms)	Low	217 ± 15	^BC^	322 ± 55	0.244 (0.10)	<0.001 (0.75)	<0.001 (0.70)
	Mod	184 ± 14	^AC^	271 ± 26			
	High	154 ± 11	^AB^	232 ± 20			
Pole angle at pole plant (°)	Low	77.4 ± 1.8		69.2 ± 2.6	0.359 (0.08)	<0.001 (0.86)	0.181 (0.12)
	Mod	78.3 ± 1.8		69.4 ± 1.8			
	High	78.7 ± 1.4		69.7 ± 1.8			
Pole angle at pole off (°)	Low	32.2 ± 1.0^bc^^*^		35.9 ± 1.1^bc^	<0.001 (0.49) F_(2,26)_ = 13		^5%^<0.001 (0.92) ^12%^<0.001 (0.94)
	Mod	29.4 ± 1.0^ac^^*^		32.4 ± 1.2^ac^			
	High	27.5 ± 1.1^ab^^*^		29.9 ± 1.1^ab^			
Perpendicular CoM displacement (cm)	Low	15.2 ± 1.3^bc^^*^		16.7 ± 1.6^bc^	<0.001 (0.78) F_(2,26)_ = 45		^5%^<0.001 (0.96) ^12%^<0.001 (0.93)
	Mod	19.2 ± 1.4^ac^		19.8 ± 1.8^ac^			
	High	24.2 ± 1.7^ab^		23.2 ± 2.1^ab^			

In case of non-sig. interaction effect, upper-case lettering indicates sig. difference from low (^A^), moderate (^B^), and high (^C^) power outputs for pooled inclines (2-way ANOVA without interaction term)

In case of sig. interaction effect, lower-case lettering indicates sig. difference from low (^a^), moderate (^b^), and high (^c^) power outputs for each specific incline (1-way ANOVA for each incline)

* indicates sig. difference from 12% incline at the same work rate

### Kinematics

**Fig 3:**
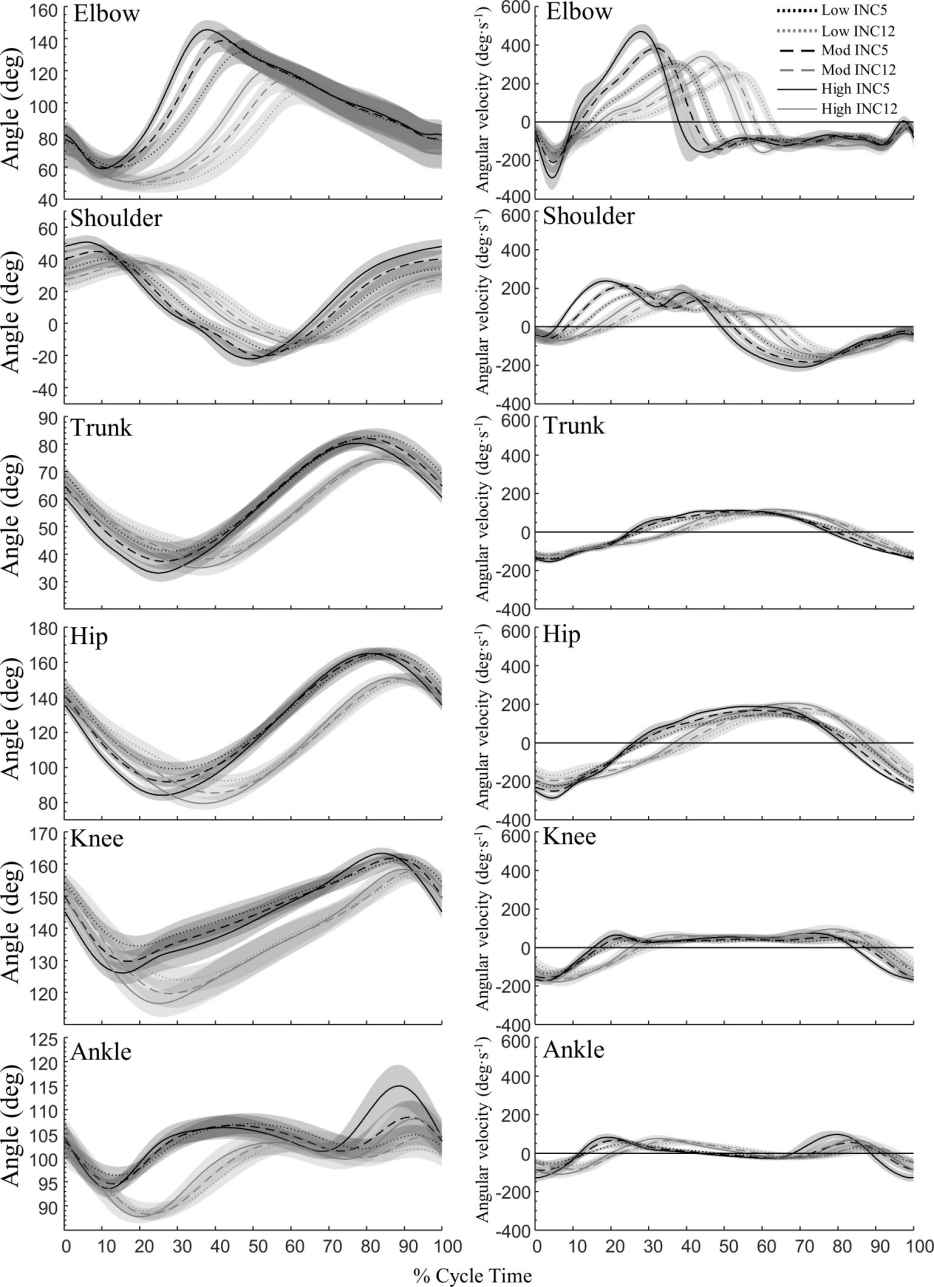
Joint angle and angular velocity against normalized cycle time during uphill DP at shallow (5%) and steep (12%) inclines. Lines are means and 95% CI is indicated by shaded area [N = 14].

Some differences in both amplitude and timing of kinematics can be seen between INC5 and INC12 in Fig 3. Elbow flexion ROM was about the same, while elbow and shoulder extension ROM were larger at INC5 (both p<0.001). Shoulder extension started earlier at INC5 than at INC12. Hip, knee, and ankle (dorsal) flexion ROM during poling was larger at INC12 compared to INC5 (both p<0.05). The maximal lower-extremity extension angles during swing were all greater at INC5. The maximal perpendicular height of CoM was higher at INC5 compared to INC12, but the minimum height of CoM was less at INC12. The within-cycle perpendicular displacement of CoM was lower at INC5 at low P_mean_, but this relationship was reversed at high P_mean_ (interaction effect p<0.001; Table 1).

### Dynamics

**Fig 4:**
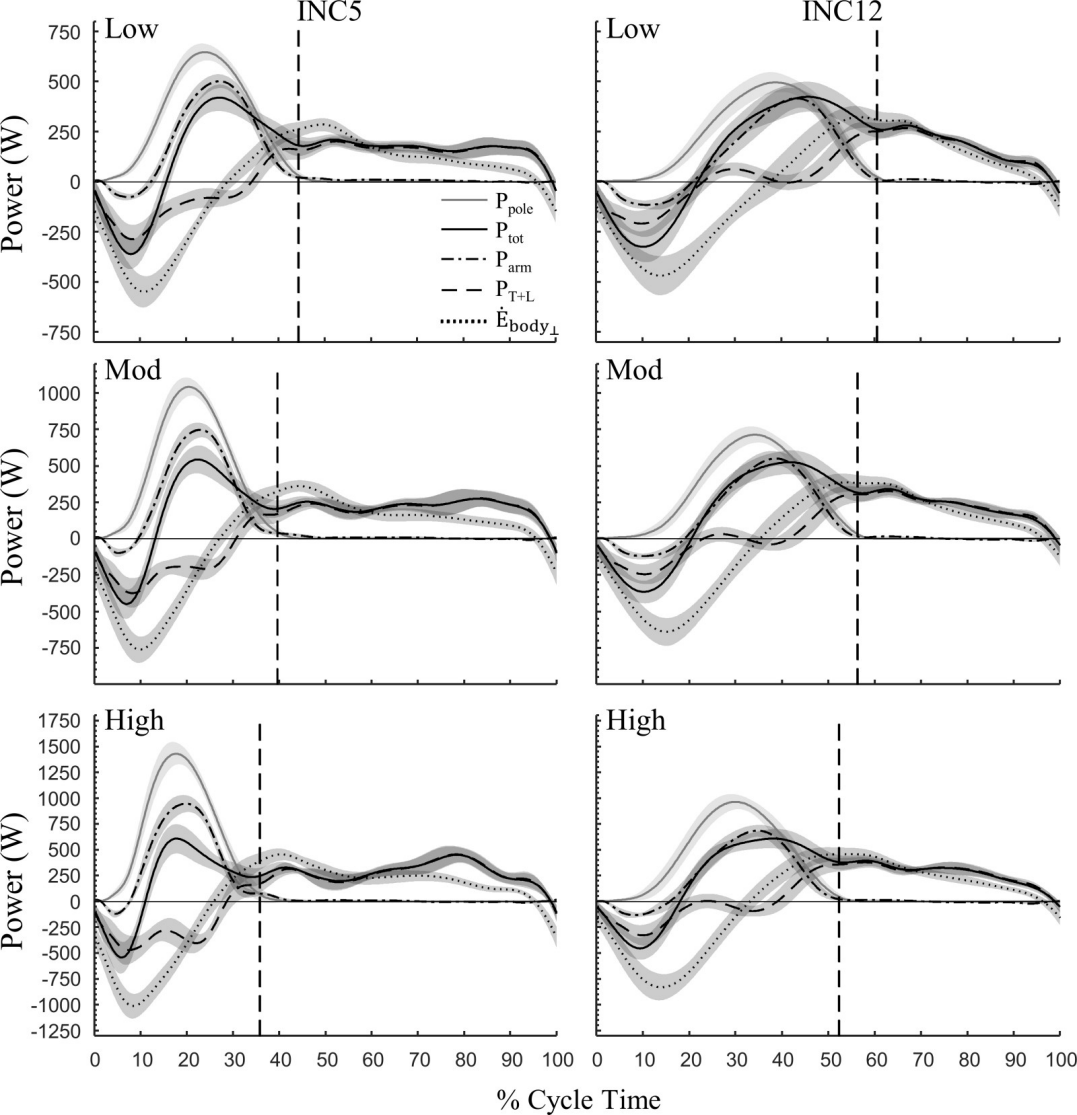
Mechanical power against normalized cycle time at shallow (INC5, 5%) and steep (INC12, 12%) inclines in uphill DP. Top, middle, and bottom panels represent low, moderate, and high external power, respectively. Lines represent mean with 95% CI indicated by shaded area [N = 14]. Pole power (P_pole_), total muscle power output (P_tot_), arm power (P_arm_), and trunk+legs power (P_T+L_). Also shown is the rate of change of mechanical energy associated with body movements perpendicular to the treadmill belt (${\dot{E}}_{{body}_{⊥}}$). The vertical dashed lines represent end of poling phase.

**Fig 5:**
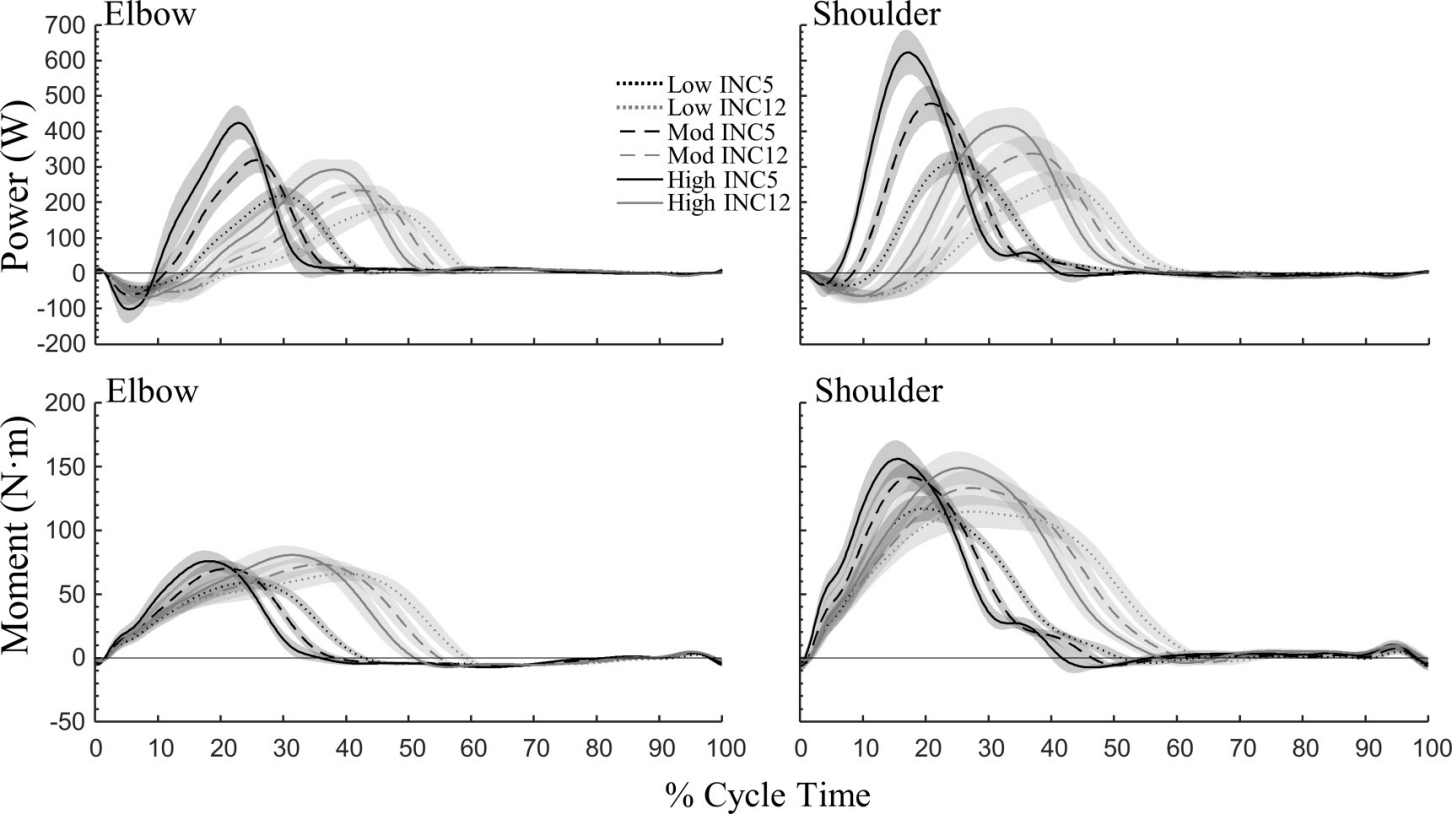
Power output and net moment about the elbow and shoulder joints in uphill DP at shallow (INC5, 5%) and steep (INC12, 12%) inclines at low, moderate, and high external power. Lines represent mean with 95% CI indicated by shaded areas [N = 14].

Fig 4 shows the behaviour of P_pole_, P_tot_, P_arm_, P_T+L_, and ${\dot{E}}_{{body}_{⊥}}$. At both inclines and all external powers, both P_arm_ and P_T+L_ were negative during the first part of propulsion, leading to negative P_tot_. As elbow and shoulder extension began, positive P_arm_ rapidly increased; however, shoulder power was higher at both inclines and all work rates, and a proximodistal sequence in power generation occurred (i.e., peak shoulder power preceded peak elbow power; Fig 5). At INC12, P_arm_ became about equal to P_pole_, while P_T+L_ fluctuated close to zero (Fig 4). At INC5, P_arm_ was slightly higher than P_tot_ while P_T+L_ was negative for most of poling. At both inclines, P_T+L_ became positive before the end of poling, corresponding approximately to the time point at which body raising began (${\dot{E}}_{{body}_{⊥}}$ becomes positive). Relative to the end of poling, this occurred earlier at INC12. Moreover, the time point at which ${\dot{E}}_{{body}_{⊥}}$ became positive coincided with peak P_pole_ at INC12, whereas at INC5 body energy was still decreasing (negative ${\dot{E}}_{{body}_{⊥}}$) at this point. Otherwise, the fluctuation of ${\dot{E}}_{{body}_{⊥}}$ was quite similar on both inclines, although the negative peak was larger at INC5 compared to INC12. Throughout swing, P_T+L_ was positive, as expected for extension of the trunk and legs.

**Fig 6:**
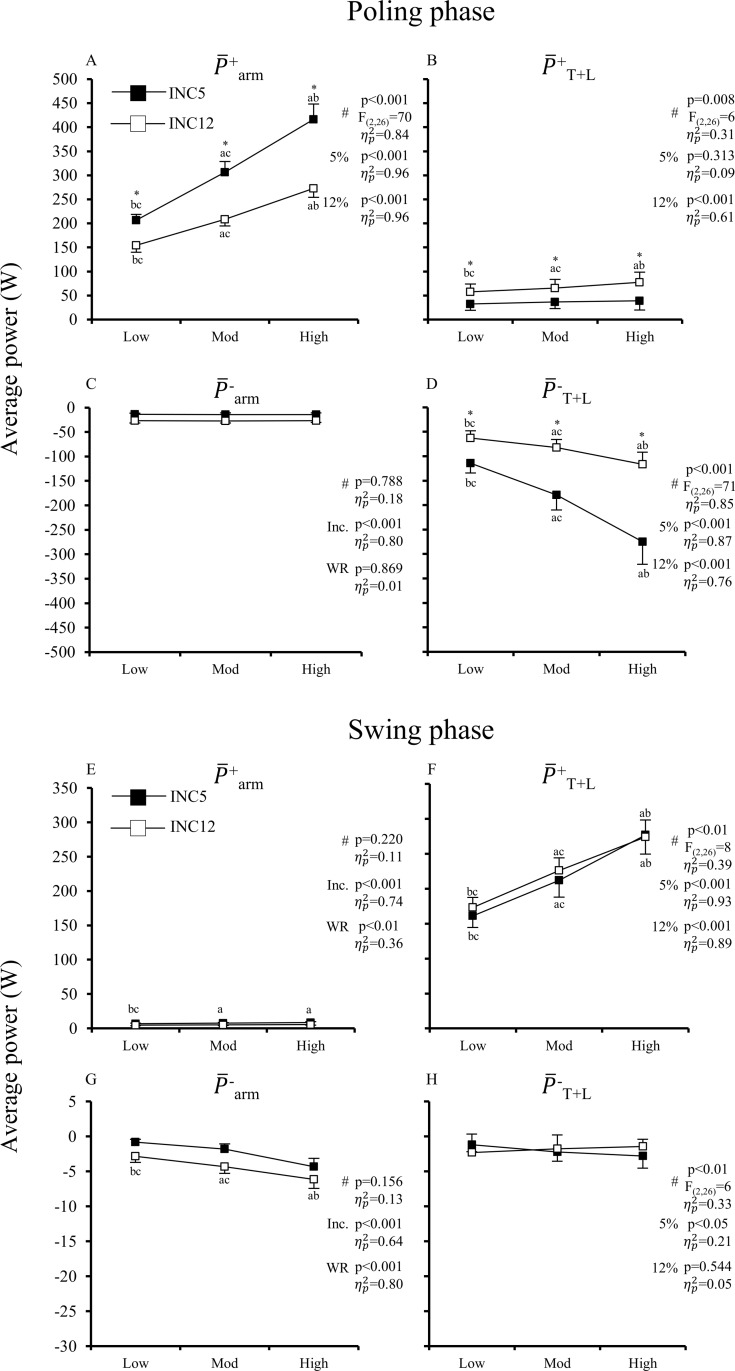
**Average positive and negative power about the arms ($\bar{P}$_arm_) and legs and trunk ($\bar{P}$_T+L_) for the poling (A-D) and swing (E-H) phases in uphill double-poling at increasing external power at shallow (INC5, 5%) and steep (INC12, 12%) inclines.** Values are means and 95% CI [N = 14]. # indicates test for interaction; if sig. interaction, p-values are shown for simple main effect for each incline (5% and 12%) while if non-sig. interaction, p-values are shown for main effect of incline (Inc.) and WR (work rate); a,b, and c indicates sig. difference from low, mod, and high work rates at each incline, respectively (p<0.05), if sig. interaction or at pooled inclines if non-sig. interaction; * indicates sig. difference between inclines at the same work rate (p<0.05).

Fig 6 shows the average positive and negative arm power and trunk+leg power during the poling and swing phases. During poling, $\bar{P}$^+^_arm_ increased considerably with work rate at both inclines, but more so at INC5 (interaction effect p<0.001, Fig 6A). $\bar{P}$^+^_T+L_ during poling was quite small, but greater at INC12 than at INC5 (Fig 6B). During poling, the amount of $\bar{P}$^-^_T+L_ became greater from low to high P_mean_, but much more so at INC5 than at INC12 (interaction effect p<0.001, Fig 6D). During swing, $\bar{P}$^+^_T+L_ increased about linearly with work rate at both inclines (Fig 6F).

**Fig 7:**
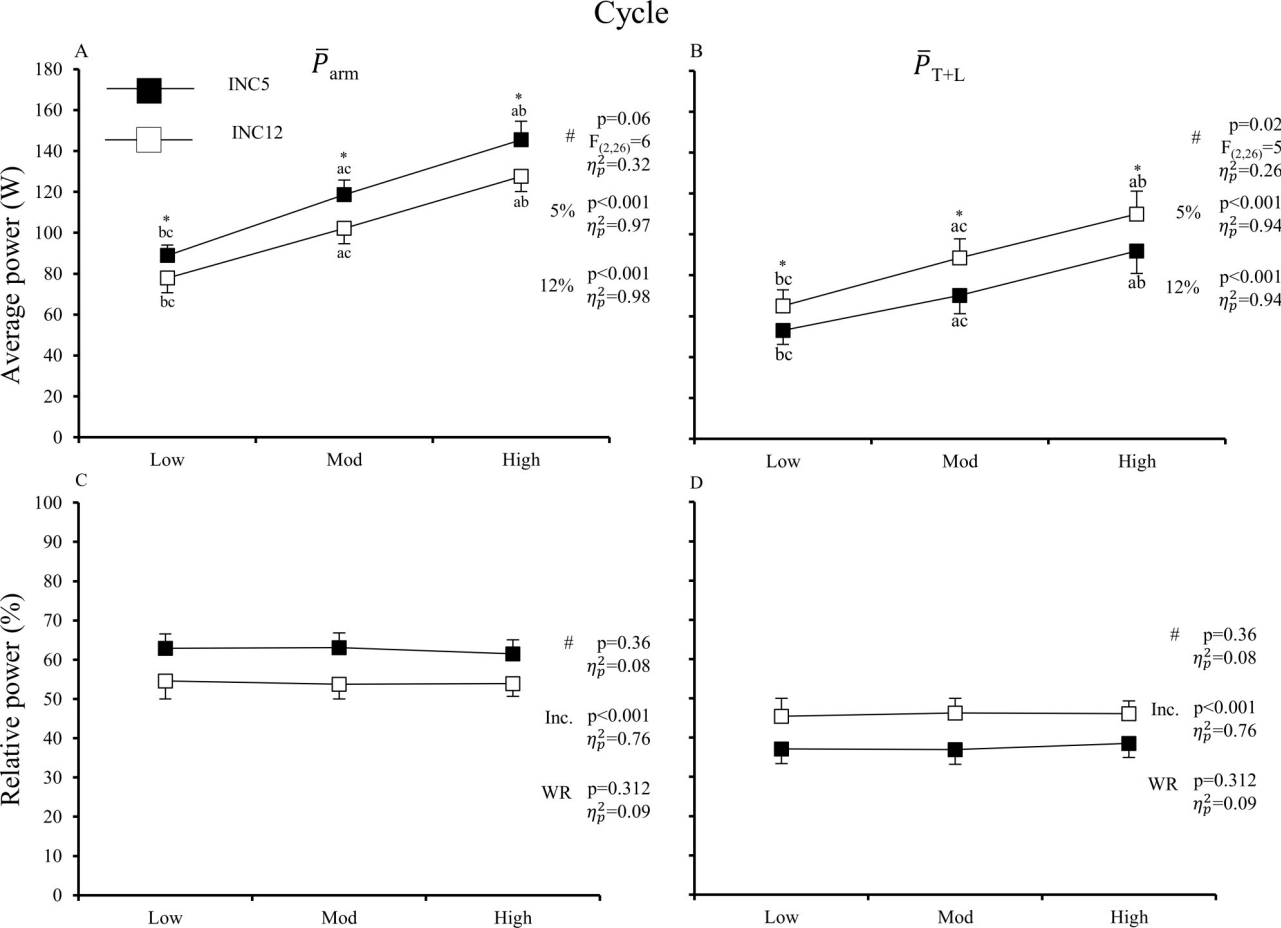
**Average absolute (A-B) and relative (C-D) arm power** ($\bar{P}$**_arm_) and leg and trunk** ($\bar{P}$**_T+L_) power over the locomotion cycle in uphill DP at increasing external power at shallow (INC5, 5%) and steep (INC12, 12%) inclines.** Values are mean and 95% CI [N = 14]. # indicates test for interaction; if sig. interaction, p-values are shown for simple main effect for each incline (5% and 12%) while if non-sig. interaction, p-values are shown for main effect of incline (Inc.) and WR (work rate); a,b, and c indicates sig. difference from low, mod, and high work rates at each incline, respectively (p<0.05); * indicates sig. difference between inclines at the same work rate (p<0.05).

Fig 7 shows the cycle average arm and trunk+leg power during poling and swing. Over the whole cycle, $\bar{P}$_arm_ was greater at INC5 than at INC12. Since both $\bar{P}$_arm_ and $\bar{P}$_T+L_ increased in a rather linear fashion, the relative contribution from $\bar{P}$_arm_ and $\bar{P}$_T+L_ (towards P_mean_) was unaffected by increasing P_mean_ at both inclines. The relative contribution from $\bar{P}$_arm_ was 63% at INC5 and reduced to 54% at INC12.

## Discussion

This study investigated the effect of incline-speed combinations on energetics and dynamics in uphill DP at shallow and steep inclines, where DP is preferred on the former but not on the latter incline. Our main question was how the relative contributions from P_arm_ and P_T+L_ are affected by different incline-speed combinations. The relative power contribution from the arms towards total external power was reduced from 63% at INC5 to 54% at INC12, and was unaffected by increasing external power at both inclines. Thus, the hypothesis of reduced use the legs as a major source of energy at steeper inclines was rejected. That is, the legs was a large source of energy at both inclines, and slightly greater at the steep incline. In general, the mechanism for transferring energy generated by the legs to propulsion power [9] was similar at both inclines: $E_{{body}_{⊥}}$ generally increased during the swing phase (positive ${\dot{E}}_{{body}_{⊥}}$) due to body raising and decreased (negative ${\dot{E}}_{{body}_{⊥}}$) due to body lowering during the poling phase, where ${\dot{E}}_{{body}_{⊥}}$ and P_pole_ were largely out-of-phase. Moreover, at INC5, the trunk and legs generated more power than likely ‘necessary’ during the swing phase, and thus considerable power was absorbed by the legs and trunk during the poling phase.

The finding that the relative contributions from P_arm_ and P_T+L_ remained essentially unaffected by increasing external power is in contrast to several previous studies showing the legs to become increasingly more involved when DP intensity is increased [10–12, 26–28], but agrees with these studies in that DP is a whole-body movement, where the legs contribute significantly to external power output. One reason for this discrepancy may be that the external power outputs of the present study were not great enough cause any essential technique alteration. It should be mentioned, however, that no previous studies have investigated the effects of external power or intensity on joint power contributions in skiing DP.

At INC12, the ‘pull’ of gravity is greater and the speed was slower compared to INC5. The skiers used different poling and swing times at these inclines to adjust for the conditions. While poling time is more directly linked to speed, the considerably shorter swing time at INC12 likely occurs to reduce speed loss in the swing phase due to gravity. Since large speed (or power) fluctuations is costly in an energetic perspective, skiers try to limit such fluctuations by increasing propulsion time or cycle rate within the given sub-technique, or by changing sub-technique altogether [6]. Skiing with higher cycle rates, however, tends to increase the metabolic cost [15]. In the present study, going from DP on a shallow to a steep incline, poling time was increased more than cycle rate, while the largest change was the shortening of swing time. Similar findings were recently shown by Stöggl and Holmberg [1] who suggested that the shorter swing times appear to be a limiting factor for DP performance on steeper inclines. In addition, the steeper incline means that the poles are forced onto the ground earlier at INC12, i.e., there is less space available for the poles to swing forward before pole plant. In contrast to level DP with, for example, enforced unnaturally high cycle rates [14], at steeper inclines the shorter swing times are brought about forcefully due to physical constraints (incline) (Fig 1). As expected, both gravity in itself and the shorter swing times led to altered body and pole positioning at INC12, especially towards the end of swing: the trunk and legs was less extended, the maximum perpendicular height of CoM was lower and occurred relatively closer in time relative to pole plant. The poles were also directed more backwards and planted closer to the feet in the fore-aft direction. These alterations all show the disappearance of the distinct preparation phase [1, 3] immediately prior to pole plant as incline increases. As a consequence, it could be hypothesized that the mechanism allowing for effective use of the legs as a major source of mechanical energy generation becomes compromised at steeper inclines, and that more power must be generated by the arms during the poling phase. However, this hypothesis was not confirmed in the present study at the current speeds and power outputs.

Considerably more arm power was generated during the poling phase at INC5 than at INC12. Still, the $\bar{P}$^+^_arm_ contribution towards $\bar{P}$_pole_ within poling was about similar at both inclines (~65% at low and ~60% at high P_mean_). Thus, the estimated contribution of $E_{{body}_{⊥}}$ to $\bar{P}$_pole_ were similar. Since most of $E_{{body}_{⊥}}$ is generated by the legs and trunk during the swing phase, it follows that similar amounts of $\bar{P}$^+^_TLE_ must be generated during swing at all inclines, which indeed happened (Fig 6F). However, although similar amounts of $\bar{P}$^+^_TLE_ were generated during the swing phase, swing time at INC12 was shorter and extension movements of the trunk and legs were reduced. At INC5, the trunk and legs had more time to perform more work at the same power. Thus, at INC5 the skiers displayed a more pronounced ‘high hip–high heel’ positioning and a more distinct preparation phase that has been deemed important for optimal generation of pole forces via the arms [7, 8, 11]. As a consequence, at INC12 the skiers shifted the body raising action forward in time. That is, a larger part of body raising occurred during late poling, which led to more $\bar{P}$^+^_T+L_ during poling. Altogether, despite expected limitations at INC12, it appears that the skiers were able to utilize the same amount (and even more) of energy from the legs and trunk as at INC5. This does not necessarily mean that the potential for leg and trunk work was the same.

One main difference between inclines was that during the poling phase, the legs and trunk absorbed more power at INC5 compared to INC12, especially when external power increased. At INC12, which typically involves smaller ski forces than at INC5, pole forces were larger and the period of high pole forces lasted longer. Thus, the poles supported more of body weight at INC12. Also, the rate of decrease in $E_{{body}_{⊥}}$, i.e., speed of body movements, was larger at INC5, especially at the end of the swing phase and into the poling phase. This suggests that at INC5 there was a larger ‘excess’ of $E_{{body}_{⊥}}(E_{{body}_{⊥}}$ not used directly for P_pole_), which likely is absorbed by the legs and trunk in a bouncing-like movement.

Overall, at INC5 more arm power was produced in total and the legs and trunk absorbed more power than at INC12. An explanation for this may be that at steeper inclines and slower speeds (e.g., INC12), large and long-lasting pole forces are typically (physically) required. In order to meet this demand, a large leg and trunk power contribution is needed. Together with the boundary conditions (in maximal body raising), this results in a reduced abundance of $E_{{body}_{⊥}}$ that subsequently needs to be absorbed. At shallower inclines and faster speeds (e.g., INC5), large and short-lasting pole forces are typically generated, but are not necessarily required, i.e., pole forces can be smaller in magnitudes but generated over a longer duration [7, 14]. Still, at both level and shallow inclines, the strategy of engaging the legs in order to generate these larger and short-lasting pole forces (and P_pole_), thereby increasing swing time, increases efficiency [8]. Although this strategy seem to create an abundance of $E_{{body}_{⊥}}$, and assuming that skilled athletes have few wasted motions, it is, however, likely the most effective strategy, especially if the ‘excessive’ energy is effectively reutilized by the legs and trunk in a stretch-shortening cycle during this bouncing-like behaviour.

Moreover, the longer poling times when DP on steeper uphill terrain likely has implications for dynamics and muscle forces in the arms. Although more power was generated by the arms at INC5 than at INC12, the magnitudes of both elbow and shoulder extensor moments were about equal, though lasted longer at INC12, both in absolute time and as a percentage of cycle time. This may lead to an increased metabolic cost of generating force (see e.g., [29, 30]), and, perhaps more importantly, increase the sense of effort [31]. Intuitively, the lesser work done by the arms on steeper inclines suggests a lower demand on the arms, i.e., the arms have an easier job at INC12 than at INC5. However, the fact that skiers perceive this reduced amount of work done by the arms at steep incline as more demanding [6] may be explained by that at steep incline the working condition for the arms become less advantageous. The longer time period of large extensor moments (and muscle forces) at INC12 suggest that the arm extensors are operating within a less favourable range of the force-length-velocity relationship. Furthermore, the shorter swing times (and thus shorter muscle relaxation times) presumably compromise muscle perfusion, leading to an unfortunate hemodynamic situation (e.g., [14]). Altogether, the way in which power was generated and absorbed within the cycle was influenced by incline.

Concerning dynamics specific to the arms, studies have hypothesized that elbow and shoulder extensors go through a stretch-shortening cycle (SSC) during DP, which has been considered a characteristic of DP performance which becomes more pronounced at faster speeds [7, 32, 33]. In the present study, the elbow and shoulder flexion-extension movements involving negative and positive power, respectively, indicate that stretch-shortening of the upper-extremity extensors may occur. However, at our shallow incline and highest speeds (INC5), the shoulder kinematics and dynamics suggest that any shoulder extensor SSC activity was diminishing rather than increasing with speed, which is opposite to the findings of e.g., Lindinger et al. [33] and Zoppirolli et al. [32]. It should be noted that these studies [32, 33] did not measure shoulder angle change or dynamics, and was based on upper-extremity muscle activity in combination with elbow kinematics only. The elbow kinematics of those studies were generally similar to both inclines in the present study. Furthermore, the patterns of shoulder and elbow power observed in the present study do not fully resemble those found recently in a study on ergometer DP [10]. In ergometer DP, the peak negative elbow power was larger and occurred simultaneously with peak positive shoulder power, indicating that power transfer mechanisms (between the body, the upper-extremity, and P_pole_) rather than SSC mechanisms may prevail in explaining the kinematics and dynamics of especially the upper extremity during DP. The different findings between ergometer and roller-skiing DP, and especially between different modes of roller-skiing DP, indicate that more research is needed. For example, it could be useful to combine dynamics analysis with muscle activity analysis to further understand more detailed joint and muscle dynamics in propulsion mechanics in different modes of DP. Future research may also compare DP dynamics between different groups of skiers, e.g., world class skiers vs. skiers of a distinctly lower performance level, or between males and females since females DP less [13, 34]. Such analysis may help elucidating more detailed but perhaps important differences in technique execution which may aid athletes and coaches in improving performance at all levels.

## Conclusion

At all external powers studied here in uphill DP, the arm power contribution was reduced from about 63% at 5% incline to about 54% at 12% incline. Thus, the relative contribution by the legs and trunk was not influenced by external power at the submaximal intensities studied here, but increases slightly from shallow to steep uphill terrain during roller-skiing DP. Although less arm power production intuitively suggests that the arms have an easier task at steeper inclines, skiers perceive this lower work done by the arms at steep incline as more demanding. This might be related to disadvantageous working condition for the arms at steep incline, and to a longer period of large upper-extremity muscle extensor moments (and muscle force). At a shallow incline, the legs do more positive work than ‘necessary’ and thus part of the excessive energy is absorbed during the poling phase (by the legs). However, the associated raising and lowering of the body seems helpful in positioning the body and poles in a favourable working condition where the arms can produce relatively high power in a short amount of time and at low perceived effort.

## Supporting information

S1 File Individual data for the current paper on mechanical energetics and dynamics in uphill double poling. (XLSX)

## References

[1] Stöggl T, Holmberg H-C. Double-poling biomechanics of elite cross-country skiers: flat versus uphill terrain. Med Sci Sports Exerc. 2016;48:1580–9. 10.1249/MSS.0000000000000943 27031747

[2] Sandbakk Ø, Holmberg H-C. A reappraisal of success factors for Olympic cross-country skiing. Int J Sports Physiol Perform. 2014;9(1):117–21. 10.1123/ijspp.2013-0373 24088346

[3] Stöggl T, Holmberg H-C. Force interaction and 3D pole movement in double poling. Scand J Med Sci Sports. 2011;21(6):e393–e404. 10.1111/j.1600-0838.2011.01324.x 21545537

[4] Welde B, Stöggl T, Mathisen GE, Supej M, Zoppirolli C, Winther AK, et al The pacing strategy and technique of male cross-country skiers with different levels of performance during a 15-km classical race. PLoS One. 2017;12(11):e0187111 10.1371/journal.pone.0187111 29117228 PMC5678876

[5] Pellegrini B, Zoppirolli C, Bortolan L, Holmberg H-C, Zamparo P, Schena F. Biomechanical and energetic determinants of technique selection in classical cross-country skiing. Hum Mov Sci. 2013;32:1415–29. 10.1016/j.humov.2013.07.010 24071549

[6] Dahl C, Sandbakk Ø, Danielsen J, Ettema G. The role of power fluctuatoins in the preference of diagonal vs. double poling sub-technique at different incline-speed combinations in elite cross-country skiers. Front Physiol. 2017 10.3389/fphys.2017.00094 28270769 PMC5318423

[7] Holmberg H-C, Lindinger S, Stöggl T, Eitzlmair E, Müller E. Biomechanical analysis of double poling in elite cross-country skiers. Med Sci Sports Exerc. 2005;37(5):807–18. 15870635 10.1249/01.mss.0000162615.47763.c8

[8] Holmberg H-C, Lindinger S, Stöggl T, Björklund G, Müller E. Contribution of the legs to double-poling performance in elite cross-country skiers. Med Sci Sports Exerc. 2006;38(10):1853–60. 10.1249/01.mss.0000230121.83641.d1 17019309

[9] Danielsen J, Sandbakk Ø, Holmberg H-C, Ettema G. Mechanical energy and propulsion in ergometer double poling by cross-country skiers. Med Sci Sports Exerc. 2015;47(12):2586–94. 10.1249/MSS.0000000000000723 26110695

[10] Danielsen J, Sandbakk Ø, McGhie D, Ettema G. The effect of exercise intensity on joint power and dynamics in ergometer double-poling performed by cross-country skiers. Hum Mov Sci. 2018;57:83–93. 10.1016/j.humov.2017.11.010 29179043

[11] Lindinger SJ, Stöggl T, Müller E, Holmberg H-C. Control of speed during the double poling technique performed by elite cross-country skiers. Med Sci Sports Exerc. 2009;41(1):210–20. 10.1249/MSS.0b013e318184f436 19092686

[12] Zoppirolli C, Pellegrini B, Modena R, Savoldelli A, Bortolan L, Schena F. Changes in upper and lower body muscle involvement at increasing double poling velocities: an ecological study. Scand J Med Sci Sports. 2017;27(11):1292–9. 10.1111/sms.12783 27726202

[13] Hegge AM, Bucher E, Ettema G, Faude O, Holmberg H-C, Sandbakk Ø. Gender differences in power production, energetic capacity and efficiency of elite cross-country skiers during whole-body, upper-body, and arm poling. Eur J Appl Physiol. 2016;116(2):291–300. 10.1007/s00421-015-3281-y 26476546

[14] Lindinger SJ, Holmberg H-C. How do elite cross-country skiers adapt to different double poling frequencies at low to high speeds? Eur J Appl Physiol. 2011;111(6):1103–19. 10.1007/s00421-010-1736-8 21113613

[15] Sandbakk Ø, Holmberg H-C, Leirdal S, Ettema G. Metabolic rate and gross efficiency at high work rates in world class and national level sprint skiers. Eur J Appl Physiol. 2010;109(3):473–81. 10.1007/s00421-010-1372-3 20151149

[16] van den Bogert AJ, de Koning JJ, editors. On optimal filtering for inverse dynamics analysis. Proceedings of the IXth biennial conference of the Canadian society for biomechanics; 1996: Simon Fraser University Vancouver.

[17] Bisseling RW, Hof A. Handling of impact forces in inverse dynamics. J Biomech. 2006;39:2438–44. 10.1016/j.jbiomech.2005.07.021 16209869

[18] de Leva P. Adjustments to Zatsiorsky-Seluyanov's segment inertia parameters. J Biomech. 1996;29(9):1223–30. 8872282 10.1016/0021-9290(95)00178-6

[19] van Ingen Schenau GJ, Cavanagh PR. Power equations in endurance sports. J Biomech. 1990;23(9):865–81. 2211732 10.1016/0021-9290(90)90352-4

[20] Winter DA. Biomechanics and Motor Control of Human Movement. 4 ed. New York (NY): John Wiley & Sons; 2009 p. 154–5.

[21] Donelan J, Kram R, Kuo A. Simultaneous positive and negative external mechanical work in human walking. J Biomech. 2005;35:117–24. 10.1016/s0021-9290(01)00169-5 11747890

[22] Elftman H. Forces and energy changes in the leg during walking. Am J Physiol. 1939;125(2):339–56.

[23] Zelik KE, Kuo AD. Mechanical work as an indirect measure of subjective costs influencing human movement. PloS one. 2012;7(2):e31143 10.1371/journal.pone.0031143 22383998 PMC3286468

[24] Schache AG, Brown NAT, Pandy MG. Modulation of work and power by the human lower-limb joints with increasing steady-state locomotion speed. J Exp Biol. 2015;218:2472–81. 10.1242/jeb.119156 26056240

[25] Farris DJ, Sawicki GS. The mechanics and energetics of human walking and running: a joint level perspective. J R Soc Interface. 2012;(9):110–8. 21613286 10.1098/rsif.2011.0182 PMC3223624

[26] Rud B, Secher N, Nilsson J, Smith G, Hallén J. Metabolic and mechanical involvement of arms and legs in simulated double pole skiing. Scand J Med Sci Sports. 2014;24(6):913–9. 10.1111/sms.12133 24151924

[27] Nilsson J, Tinmark F, Halvorsen K, Arndt A. Kinematic, kinetic and electromyographic adaptation to speed and resistance in double poling cross country skiing. Eur J Appl Physiol. 2013;113(6):1385–94. 10.1007/s00421-012-2568-5 23229884

[28] Bojsen-Møller J, Losnegard T, Kemppainen J, Viljanen T, Kalliokoski KK, Hallén J. Muscle use during double poling evaluated by positron emission tomography. J Appl Biomech. 2010;109(6):1895–903. 10.1152/japplphysiol.00671.2010 20947710

[29] Griffin T, Roberts T, Kram R. Metabolic cost of generating muscular force in human walking: insights from load-carrying and speed experiments. J Appl Physiol. 1985;95(1):172–83. 10.1152/japplphysiol.00944.2002 12794096

[30] Roberts T, Kram R, Weyand P, Taylor C. Energetics of bipedal running. I. Metabolic cost of generating force. J Exp Biol. 1998;201:2745–51. 9732329 10.1242/jeb.201.19.2745

[31] Prilutsky BI, Gregor RJ. Swing-and support-related muscle actions differentially trigger human walk-run and run-walk transitions. J Exp Biol. 2001;204:2277–87. 11507111 10.1242/jeb.204.13.2277

[32] Zoppirolli C, Holmberg H-C, Pellegrini B, Quaglia D, Bortolan L, Schena F. The effectiveness of stretch–shortening cycling in upper-limb extensor muscles during elite cross-country skiing with the double-poling technique. J Electromyogr Kinesiol. 2013;23(6):1512–9. 10.1016/j.jelekin.2013.08.013 24064180

[33] Lindinger SJ, Holmberg H-C, Müller E, Rapp W. Changes in upper body muscle activity with increasing double poling velocities in elite cross-country skiing. Eur J Appl Physiol. 2009;106(3):353–63. 10.1007/s00421-009-1018-5 19280214

[34] Solli GS, Kocbach J, Seeberg TM, Tjønnås J, Rindal OMH, Haugnes P, et al Sex-based differences in speed, sub-technique selection, and kinematic patterns during low- and high-intensity training for classical cross-country skiing. PloS one. 2018;13(11):e0207195 10.1371/journal.pone.0207195 . 30440017 PMC6237352

